# A case of spontaneous colonic perforation in collagenous colitis

**DOI:** 10.1186/s40792-019-0647-0

**Published:** 2019-05-31

**Authors:** Haruki Mori, Toru Miyake, Tomoharu Shimizu, Tsuyoshi Yamaguchi, Sachiko Kaida, Katsushi Takebayashi, Hiroya Iida, Akinori Otsuki, Osamu Inatomi, Katsuyuki Kitoh, Akira Andoh, Masaji Tani

**Affiliations:** 1 Department of Surgery, Shiga University of Medical Science, Setatsukinowa-chou, Otsu, Shiga 520-2192 Japan; 2 Otsu, Japan to Setatsukinowa-chou, Otsu, Shiga 520-2192 Japan

**Keywords:** Colonic perforation, Collagenous colitis, Spontaneous

## Abstract

**Background:**

Collagenous colitis (CC) is a clinicopathologic syndrome characterized by chronic watery diarrhea and distinctive histopathologic features. Spontaneous perforation of CC is extremely rare, because CC is usually managed medically, and the need for surgical intervention is rare. We report a surgical case of spontaneous colonic perforation of CC with acute abdomen disease.

**Case presentation:**

A 77-year-old man was admitted to our hospital for abdominal pain and watery diarrhea. Computed tomography (CT) showed a thickened bowel wall with edema involving free air around the splenic flexure of the colon. Therefore, we performed emergency surgery with a diagnosis of colonic perforation. Intraoperative findings revealed colonic necrosis at the splenic flexure, so we performed a left hemicolectomy. Histopathological examination revealed typical findings of CC, a thick subepithelial collagenous band and deep ulcers with perforation. The postoperative course was uneventful, and the patient was discharged on the 28th postoperative day. After changing the proton pump inhibitor (PPI) from lansoprazole (LPZ) to rabeprazole (RPZ), he has not complained of diarrhea symptoms.

**Conclusions:**

Although spontaneous perforation is a rare complication of CC, it is possible to be diagnosed by symptom of acute abdomen disease. This is the seventh case of spontaneous colonic perforation of CC worldwide.

## Background

Collagenous colitis (CC) is characterized by chronic diarrhea and usually occurs in middle-aged women. Endoscopic examinations are generally unremarkable [1], laboratory and radiological test results are usually normal, and stool cultures are sterile in many cases. Thus, there is not a reliable biomarker for CC diagnosis.

Endoscopic evaluation of the colon is often normal, but erythema, edema, changes in blood vessels, and mucosal clefts may be observed [2]. Definitive diagnosis is established by the pathological diagnosis, an endoscopic biopsy revealing a thickened subepithelial collagenous band (10–30 μm) found in the wall of the colon with epithelial damage and chronic inflammation of the lamina propria [3].

CC is a heterogeneous disorder and may have multiple etiologies making pathogenesis unclear. Several medications have been implicated as contributing factors, notably aspirin and other non-steroidal anti-inflammatory drugs (NSAIDs), histamine 2 receptor blockers, and proton pump inhibitors, such as lansoprazole and certain selective serotonin reuptake inhibitors [4]. Autoimmune conditions, including rheumatoid arthritis, thyroid disorders, ulcerative colitis, and celiac disease, are also associated with colitis. Furthermore, it has been reported that smoking and bacterial infections are associated with CC. But it is unclear that the family history and genetic abnormalities are risk factors for CC [5, 6]. CC is rarely associated with serious complications; however, spontaneous and post-colonoscopic perforated cases have been reported [3]. Mechanisms of rare spontaneous perforations in patients with CC remain unclear. We report herein an interesting and rare case of the above.

## Case presentation

**Fig. 1:**
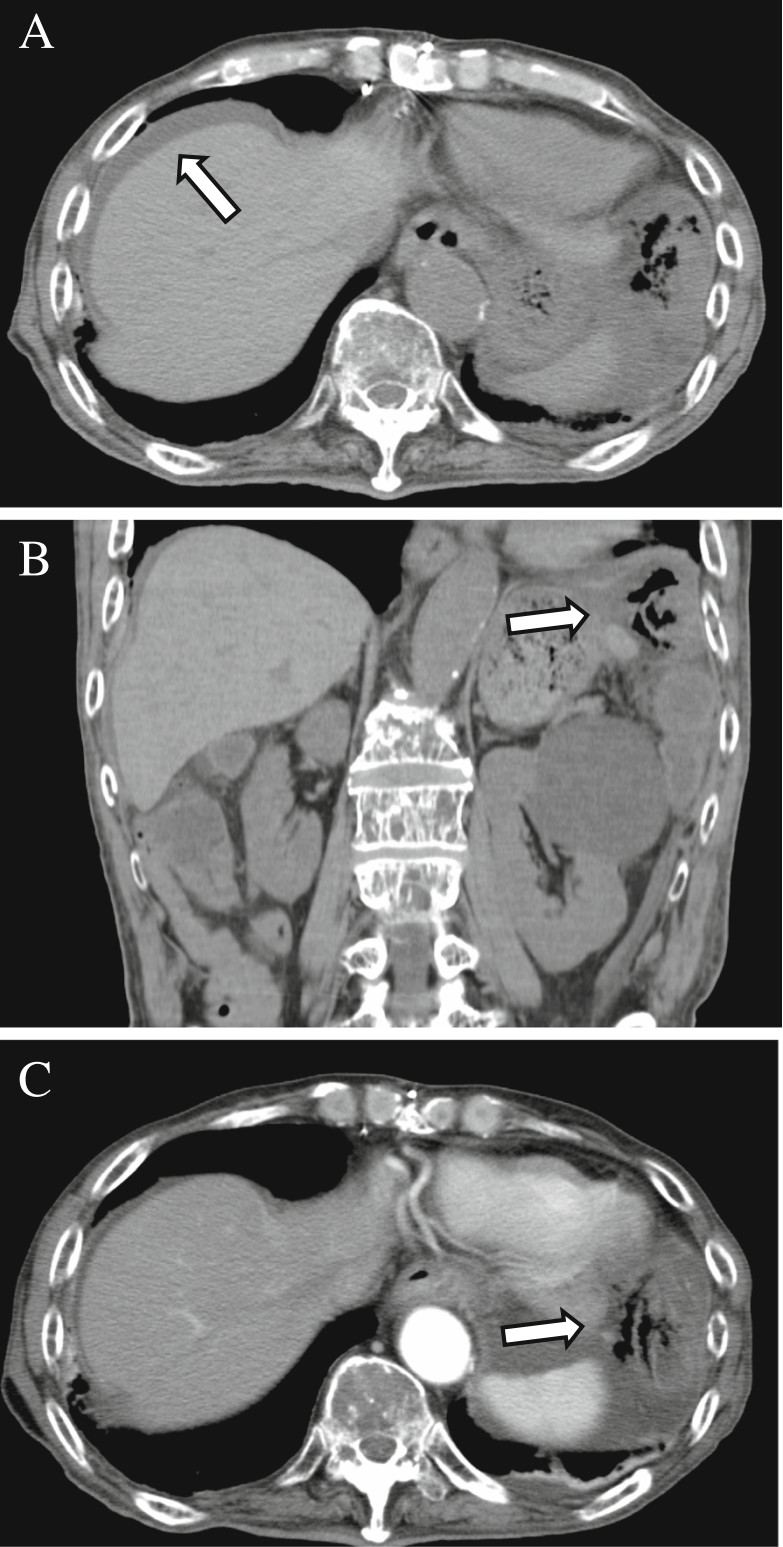
Abdominal computed tomography (CT). **a** Ascites around the liver (arrow). **b**, **c** Thickness in bowel wall and involving the free air around the colonic splenic flexure (arrow)

**Fig. 2:**
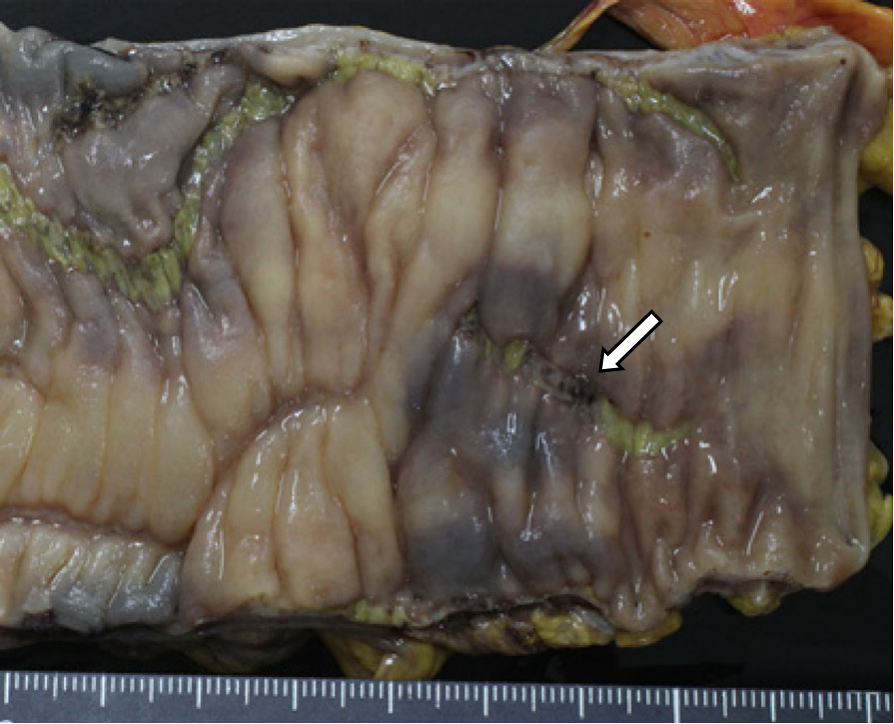
Macroscopy of the resected colon: cross sections of the bowel show normal-appearing mucosa, markedly thickened edematous wall, and longitudinal ulcer. The arrow indicates the perforation site

**Fig. 3:**
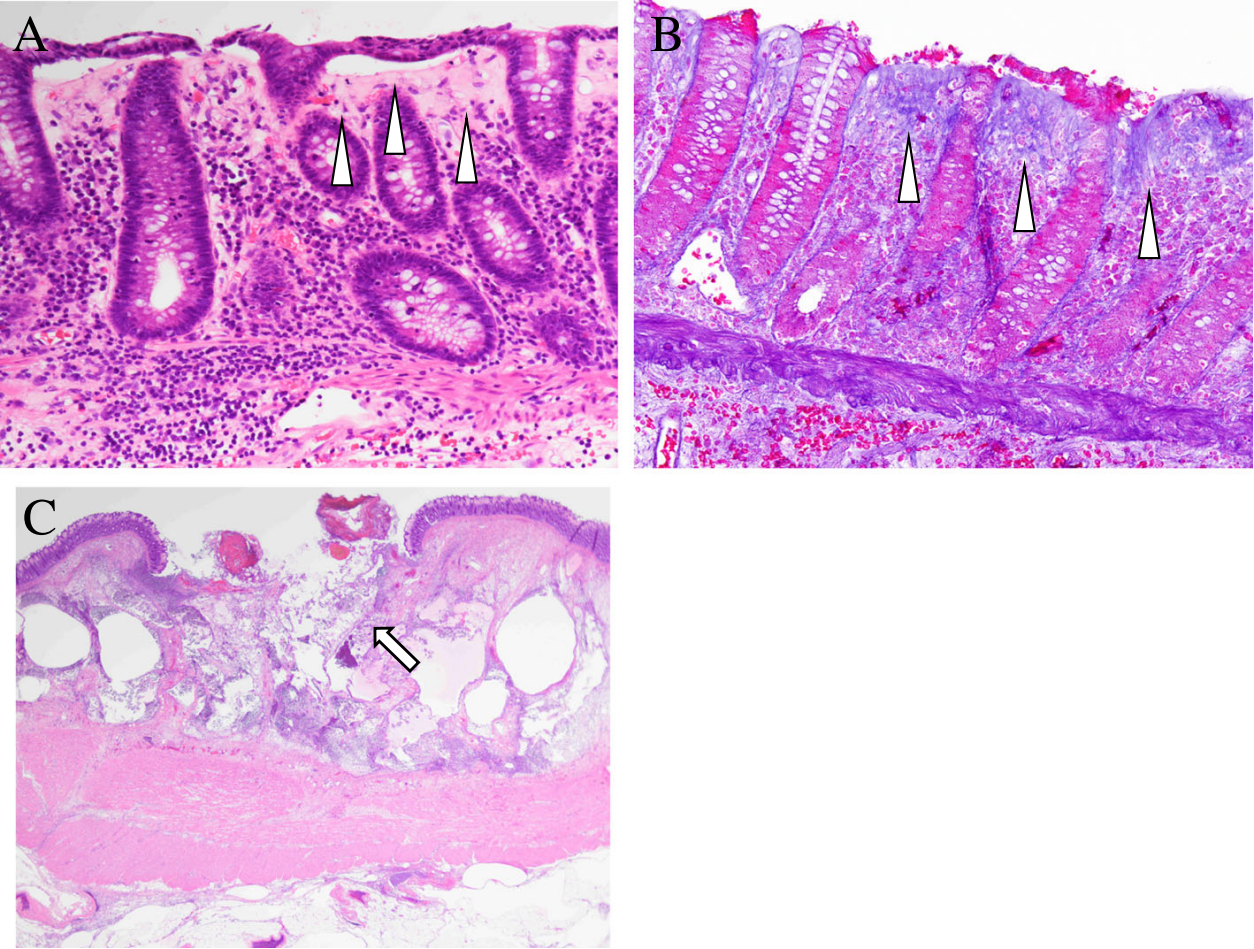
Histological examination. **a** Typical findings of collagenous colitis with a thick subepithelial collagenous band (arrowhead). **b** Collagenous band was stained by Azan (arrowhead). **c** Ulcerated area with perforation (arrow)

A 77-year-old man was admitted to Shiga University of Medical Science (SUMS) Hospital complaining of abdominal pain and frequent episodes of non-bloody watery diarrhea, lasting for 2 months. His past medical history included a gastric ulcer 40 years earlier, hypertension, and chemotherapy for multiple myeloma. His current medications were aspirin, prednisolone, melphalan, and lansoprazole (LPZ). His *body* temperature was within the normal range. Physical examination revealed acute left abdominal pain and muscular defense. Laboratory results revealed a white blood cell count of 2100/μl (normal range, 3000–8000/μl), and C-reactive protein (CRP) level was 0.19 mg/dl (normal range, < 0.30 mg/dl). Computed tomography (CT) showed a thickened bowel wall with edema involving free air around the colonic splenic flexure, and ascites was found on the liver surface (Fig. 1a, b). The patient was diagnosed as having peritonitis with colonic perforation. Emergency laparotomy was performed, and it was observed that the ascites contained intestinal fluid. The colon around the splenic angle was necrotic and edematous. We performed a left hemicolectomy. Macroscopic findings (Fig. 2) showed edematous mucosa and tortuous longitudinal ulcer. Histopathological examination (Fig. 3) revealed typical findings of CC, with a thick subepithelial collagenous band and deep ulcers with perforation. Active lymphocyte infiltration was observed in all layers of the colon. There was no evidence of acute ischemic colitis or inflammatory bowel disease.

Postoperative course was uneventful, and the patient was discharged on the 28th postoperative day. PPI-induced CC was suspected due to his past history; therefore, the PPI was subsequently changed from LPZ to RPZ. Following this change, he noted an improvement in diarrhea symptoms.

## Discussion

CC is a relatively uncommon, but increasingly diagnosed form of microscopic colitis. CC was described in 2 independent reports in 1976 from Canada and Sweden [7, 8]. Patients with CC typically complain of chronic, non-bloody, watery diarrhea. It is pathologically diagnosed by the presence of increased intraepithelial lymphocytes, mixed inflammatory cells in the lamina propria, and pathognomonic appearance of a thickened subepithelial collagen band [5]. CC is usually treated successfully with medication; therefore, the need for surgical intervention is rare [9]. However, emergent surgery is necessary if there is perforation of the bowel tract, which is accompanied by collagen deposition under the mucosal epithelium that reduces intestinal elasticity and extensibility [3]. A colonoscopy or barium enema sometimes can cause colonic perforations in collagenous colitis, and these iatrogenic perforations are thought to occur secondary to mechanical trauma or luminal insufflation causing linear mucosal tears that lead to rupture [10, 11].

Only 6 patients have been reported to have a spontaneous perforation in CC (Table 1) [9, 10, 12–15]. In all cases, including ours, there was a history of non-bloody diarrhea and no previous diagnosis of CC. CC occurs more frequently in females, and all previous reports described females, but our patient was male. All patients recovered following resection of the perforated segment. Of note, all CC perforations occurred in the left colon, in contrast to a perforation after endoscopic examination, which is commonly on the right side [16–18]. In recent years, reports of cases with characteristic longitudinal ulcers expressed as “mucosal tears” or “linear mucosal defect” are increasing [19–22]. This longitudinal ulcer is elongated and presents a mucosal split form and the boundary is clear, edema and redness of the ulcer margin are poor, and therefore different from a longitudinal ulcer seen in ischemic colitis or Crohn’s disease. A tortuous, longitudinal ulcer, characteristic of CC, was also observed in our case.

**Table 1:** Reported cases of spontaneous perforation in collagenous colitis

	Author	Year	Age/sex	Medications	Perforation site	Surgery	Outcome
1	Freeman [6]	2001	37/F	None recorded	Sigmoid	Laparotomy	Alive
2	Bohr [4]	2005	56/F	Clomipramine	Splenic angle/descending colon	Segmental resection	Alive
3	Bennett [7]	2013	67/F	Aspirin, loperamide	Splenic angle	Partial colectomy	Alive
4	Akamoto [8]	2014	64/F	None	Descending colon	Left colectomy	Alive
5	Cottreau [9]	2016	49/F	Dexilansoprazole, buproprion, clonazepam, ranitidine	Descending colon	Left colectomy	Alive
6	Mitchell [5]	2016	80/F	Loperamide, levothyroxine	Splenic angle	Segmental resection	Alive
7	Our case	2018	77/M	Lansoprazole, aspirin, prednisolone, melphalan	Splenic angle	Partial colectomy	Alive

*F* female, *M* male

Although the precise mechanism is unknown for the cause of CC, previous articles report that CC is caused by genetic factors [23, 24], intestinal factors due to malabsorption of bile acid [25, 26], and drugs [27]. In recent years, CC related to LPZ has attracted attention [28]. Oral administration rate of LPZ in patients with CC is as high as 53 to 83% in Japan [29, 30], while as low as 8% in European and American reports [31].

Proton pumps exist in colonic epithelial cells. Therefore, a proton pump inhibitor is presumed to change the composition and pH values of secretions from colonic mucosa and affect the immune response, which is a possible mechanism in developing CC [32]. LPZ is mainly metabolized by Cytochrome P450 2C19 (CYP2C19) and Cytochrome P450 3A4 (CYP3A4). A genetic polymorphism of CYP2C19 is reported in 18 to 23% of the Japanese population, compared with 1 to 6% in the Western population. This fact is considered as one of the reasons that LPZ-induced CC cases are more frequent in Japan [33]. Rabeprazole (RPZ), however, is not metabolized via CYP2C19 and CC induced by RPZ is rarer than LPZ. Therefore, it is suggested that the different PPI pharmacological action is involved in the pathogenesis of CC [30]. NSAIDs, antihypertensive drugs, and hyperlipidemic drugs are also metabolized by CYP3A4. When these drugs are used in combination with LPZ, CC may develop due to the high blood concentration of LPZ and drug interaction via CYP families [34]. In our case, the patient was taking LPZ because of the history of a gastric ulcer. After surgery, his PPI was subsequently changed from LPZ to RPZ with no changes in his other. After this change, he noted an improvement in his diarrhea symptoms. Therefore, we suggest that CC of this patient seemed to be caused by LPZ.

## Conclusion

In summary, we report a case of CC triggered by a PPI. It is important to note that spontaneous perforation of CC is a possible complication of diagnosed acute abdomen disease.

## Data Availability

Not applicable.

## Abbreviations

CC: Collagenous colitis
CRP: C-reactive protein
CT: Computed tomography
CYP2C19: Cytochrome P450 2C19
CYP3A4: Cytochrome P450 3A4
LPZ: Lansoprazole
NSAIDs: Non-steroidal anti-inflammatory drugs
PPI: Proton pump inhibitor
RPZ: Rabeprazole
